# Thymic Asthma

**Published:** 1846-02

**Authors:** 


### PRACTICAL MEDICINE, &c.

Thymic Asthma.—This disease *(real or supposed)* was first fully brought before the profession by Dr. Kopp of Germany, and numerous cases, supposed to be dependant upon the enlargement of the thymus gland, have since been published in Europe and America.

Doubts have from time to time been expressed in regard to the existence of a "new disease," depending upon this cause, and these seemed to have gained strength, so that the better opinion is perhaps opposed to its existence. The article given below, from the high authority of Trousseau, in all points of pathology of infantile diseases, will go far to confirm this view, which, however, had already been expressed in similar terms by Charles A. Lee, M. D., of New York, in an article in the American Journal of Medical Sciences, for January, 1842. The following was his conclusion from the analysis of a large number of cases, "that 'thymic asthma' and 'spasm of the glottis,' are the same disease which often owes its origin to irritation of the nerves of the glottis, sometimes involving the whole excito-motary system, chiefly by teething, through the fifth pair of nerves."

*On Thymic Asthma.* By M. TROUSSEAU.—A great deal has been written of late in Germany, says M. Trousseau, on *thymic asthma*—a disease first described a few years ago by Mr. Hood, of Kilmarnock. In this "newly-discovered" disease, the thymus gland is stated to give rise to convulsions and sudden death in infants by its enlargement. The existence of such an affection was from the first questioned by the French pathologists, and M. Trousseau now states that his researches have proved to him, in the most satisfactory manner, that there is no such disease. The facts brought forward by the German physicians must be admitted, he states, but the interpretation which they give of these facts is erroneous. Instead of being instances of an undescribed form of disease, they are merely illustrations of *partial convulsions.* The analysis of the phenomena of convulsions in children has proved to M. Trousseau that such is the real nature of the cases narrated by Kopp and other physicians as examples of thymic asthma, as well as, partly, of others described under the name of *laryngismus stridulus,* or *acute asthma of Millar.* The following is a brief analysis of M. Trousseau's views on this subject:—

In children, convulsions *(éclampsie)* generally present the epileptic form. The child screams, becomes stiff, twists its body, the thorax being fixed and the respiration suspended. The face, at first pale, becomes violet; the veins are distended; then follow clonic spasms, at first rapid, then slow; after which a deep expiration and general muscular relaxation close the fit, leaving more or less somnolence and stupor. The attack lasts one or two minutes. One paroxysm may be followed nearly immediately by another; indeed, they may succeed each other indefinitely, constituting an "état de mal." But when this is the case, the convulsions are not continuous, although sometimes considered so. They may, however, be continuous, and last for hours, or even days. When this is the case, the attack is often ushered in by an epileptic paroxysm, as above; but the spasms, instead of ceasing, are repeated every second, or at very short intervals. The convulsions are continuous, because there is never any complete cessation, nor the deep stupor which follows an ordinary paroxysm. In this form of convulsion, the child, although convulsed, does not lose all consciousness—an important feature in the disease. He cries to express a want or to complain of a pain, and is able to withdraw his hand when it is pinched or tickled. The convulsion is not, therefore, as universal as it appears; it is, rigorously speaking, *partial.*

Convulsions may be still further localized. After a severe epileptic attack, one-half of the body may remain for some hours affected with clonic spasmodic motions, and yet the intellect of the child be clear, and the motions of the other side of the body harmonious.

The convulsions hitherto described are easily recognized; but convulsions may be internal as well as partial, and then they are by no means so easy to appreciate; then, also, it is that difference of opinion as to the interpretation of the symptoms begins to be entertained. Internal convulsions are partial convulsions, occupying more particularly the muscles of the globe of the eye, of the pharynx, of the larynx, and of the apparatus of respiration. The most ordinary form of internal convulsion is characterized by turning of the globe of the eye with mobility, nearly total loss of consciousness, or, at least, a certain amount of stupor, extreme difficulty or impossibility of deglutition, and by respiration, uneven, sometimes scarcely perceptible, sometimes deep and blowing—in a word, by an attenuation of most of the phenomena of epilepsy, and by the absence of the violent convulsions of the limbs and face.

Sometimes the diaphragm and the inspiratory muscles of the abdomen and of the chest alone act, and then, for one, two or three minutes, a peculiar laryngeal blowing sound is heard, as if there existed an obstacle to the entrance and to the exit of the air. If the proper muscles of the larynx are at the same time convulsed, as their motions do not coincide, the disordered condition of the respiration appears alarming, although it is only really so when this state is much prolonged. Such is the real explanation of those states of disordered respiration which have been called thymic asthma, or laryngismus stridulus. A want of harmony between the spasmodic motions of the diaphragm, and of the muscles which move the arytænoid cartilages, is sufficient to produce the laryngeal sibilus, the orthopnœa. In the regular act of inspiration, the superior part of the larynx opens at the same time that the diaphragm descends, and produces a vacuum in the chest. If the contraction of the diaphragm takes place too rapidly, and if, at the same time, there is spasm of the larynx, as in whooping-cough, the inspiration becomes nearly impossible, and is accompanied by a violent sibilus. In the case which we are examining, however, it is not necessary to call to our assistance a want of harmony between the movements of the diaphragm and those of the muscles of the larynx; it is sufficient to suppose that the will or the instinct no longer presides, for a moment, over the movements of the arytænoidean cartilages; the muscles which move them, no longer obeying any nervous impulsion, are for the time in the condition of those of animals in whom the recurrent laryngeal nerve has been divided.

The above details explain how it is that thymic asthma, so frequent in the eyes of some observers, is never found by others. The former attribute to an increase in size of the thymus, accompanied by paroxistic accidents, what the latter consider to be merely one of the forms of convulsions in children. The thymus, like the supra-renal capsules, is an organ of transition, destined to become atrophied after the birth of the human fœtus, and less than any other organ likely to be hypertrophied. During the six years that M. Trousseau has been at the head of important wards for very young children, *he has not once met with the the thymus gland sufficiently enlarged to give rise to the slightest accident.*

M. Trousseau concludes his essay by promising, in a future article, to point out the connection which exists between convulsions and laryngismus stridulus and the acute asthma of children. At the same time he thinks it right to state that these diseases are not mere forms of infantile convulsions, as is the case with thymic asthma.—*Lancet*, Aug. 30, from *Jour. de Méd. in Am. Jour.*

